# A new quantitative 3D gap area measurement of fracture displacement of intra-articular distal radius fractures: Reliability and clinical applicability

**DOI:** 10.1371/journal.pone.0275206

**Published:** 2022-09-27

**Authors:** Lisanne J. M. Roelofs, Anne M. L. Meesters, Nick Assink, Joep Kraeima, Tim D. Van der Meulen, Job N. Doornberg, Jean-Paul P. M. De Vries, Joost Hoekstra, Kaj ten Duis, Frank F. A. IJpma

**Affiliations:** 1 Department of Surgery, Subdivision of Trauma Surgery, University Medical Center Groningen, University of Groningen, Groningen, The Netherlands; 2 3D Lab/Department of Oral and Maxillofacial Surgery, University Medical Center Groningen, Groningen, The Netherlands; 3 Department of Surgery, Subdivision of Orthopedic Surgery, University Medical Center Groningen, University of Groningen, Groningen, The Netherlands; 4 Department of Surgery, University Medical Center Groningen, University of Groningen, Groningen, The Netherlands; PLOS: Public Library of Science, UNITED KINGDOM

## Abstract

**Introduction:**

Gap and step-off measurements are generally used in the surgical decision-making process of distal radius fractures. Unfortunately, there is no consensus on treatment choice as these measurements are prone to inter- and intraobserver variability. In this study, we aim to introduce a new 3D fracture quantification method and compare it to conventional fracture analysis.

**Methods:**

Forty patients with a minimally displaced intra-articular distal radius fracture that was treated nonoperatively between 2008–2015 were included. 2D-CT images were reassessed by three orthopedic trauma surgeons who performed gap and step-off measurements. Subsequently, 3D models were created and a 3D measurement method for fracture displacement was developed. For each fracture, the ‘3D gap area’ (3D surface between all fracture fragments) was determined by three observers. Interobserver agreements were calculated for all measurements, and the intraobserver agreement was calculated for the new 3D measurement. All patients completed two questionnaires in order to link our measurements to functional outcome.

**Results:**

The interobserver agreement of the 2D measurements was fair (ICC = 0.54) for the gap and poor (ICC = 0.21) for the step-off. The median gap was 2.8 (IQR: 1.9–3.5) mm and step-off was 0.9 (IQR: 0.0–1.6) mm. Interobserver agreement on 3D gap area measurements was excellent (ICC = 0.81), with a median difference between measurements of 6.0 (IQR: 2.0–19.0) mm^2^, which indicates reliable assessment of 3D fracture displacement. Intraobserver agreement was also excellent (ICC = 0.98), with a median difference of 4.0 (IQR: 1.5–5.5) mm^2^. No significant differences in clinical outcome were found between the above and below 2mm displacement groups. The score of the DASH was 3.4 (IQR: 0.4–8.8) versus 4.2 (IQR: 0.0–11.6) respectively. Results from the PRWE questionnaire shows a similar result of 3.5 (IQR: 0.0–12.6) versus 5.0 (IQR: 0.0–25.5).

**Conclusion:**

3D gap area is a more objective measurement method compared to the conventional gap and step-off measurements to quantify the level of fracture displacement of distal radius fractures. 3D fracture assessment can be used in addition to the currently used classification systems of distal radius fractures.

## Introduction

Fractures of the distal radius are the most common type of fracture within the adult population [1,2]. Fracture diagnostics at the time of injury is based on lateral and postero-anterior X-rays of the wrist. In case of involvement of the articular surface, an additional CT-scan is often performed, which enables more detailed fracture analysis [1,3]. Conventionally, imaging-based measurements of fracture displacement are used to guide surgical decision-making. Among these are gap and step-off measurements on single CT-slices, which quantify the intra-articular incongruency. There is general consensus that operative treatment is indicated when the gap and/or step-off exceeds 2 millimeters (mm) in order to avoid unsatisfactory patient-reported outcome due to progressive osteoarthritis at long-term follow-up [4].

Unfortunately, the gap and step-off measure does not enhance uniformity in treatment choice. The method is prone to high inter- and intraobserver variability, especially in patients with minimally displaced fractures [1,5–8]. Moreover, conventional 2D-CT slices are often insufficient to display the whole extent of complex intra-articular fractures [9–13]. In the last two decades, there is a trend towards fracture classification and analysis based on 3D models [14–18]. Recent studies showed that 3D fracture imaging modalities depict the true extent of the fracture and are less prone to intra- and interobserver variability [17–19]. The use of 3D measurements and quantification can provide the physician with additional insights into the degree of fracture displacement, which might be helpful in patients in which controversy exist about the optimal choice of treatment [5,18].

Despite increasing interest in a more extensive fracture analysis method, no uniform measurement method is available to quantify fracture displacement in distal radius fractures. As we encountered several issues with conventional 2D imaging modalities, we aim to introduce and validate a 3D-CT measurement method for the analysis of intra-articular distal radius fractures. We hypothesize that a quantitative 3D distal radial fracture displacement measurement tool will improve the inter- and intraobserver agreement.

## Methods

### Patients

A diagnostic imaging study was performed in forty patients with a minimally displaced intra-articular distal radius fracture (AO classification 23B and 23C). All patients received nonoperative treatment for their distal radius fracture between 2008 and 2015 in a level 1 trauma center. Cases were selected from our distal radius fracture database provided that they were on the cutting edge of conservative or operative treatment as determined by two trauma surgeons (KtD, FIJ). Patients were only included in case a CT-scan with a ≤1 mm slice thickness was available.

This study was approved by the institutional board of the UMCG and the local medical ethical committee (research number: 201800411). Written informed consent was obtained from all participants.

### 3D models

**Fig 1:**
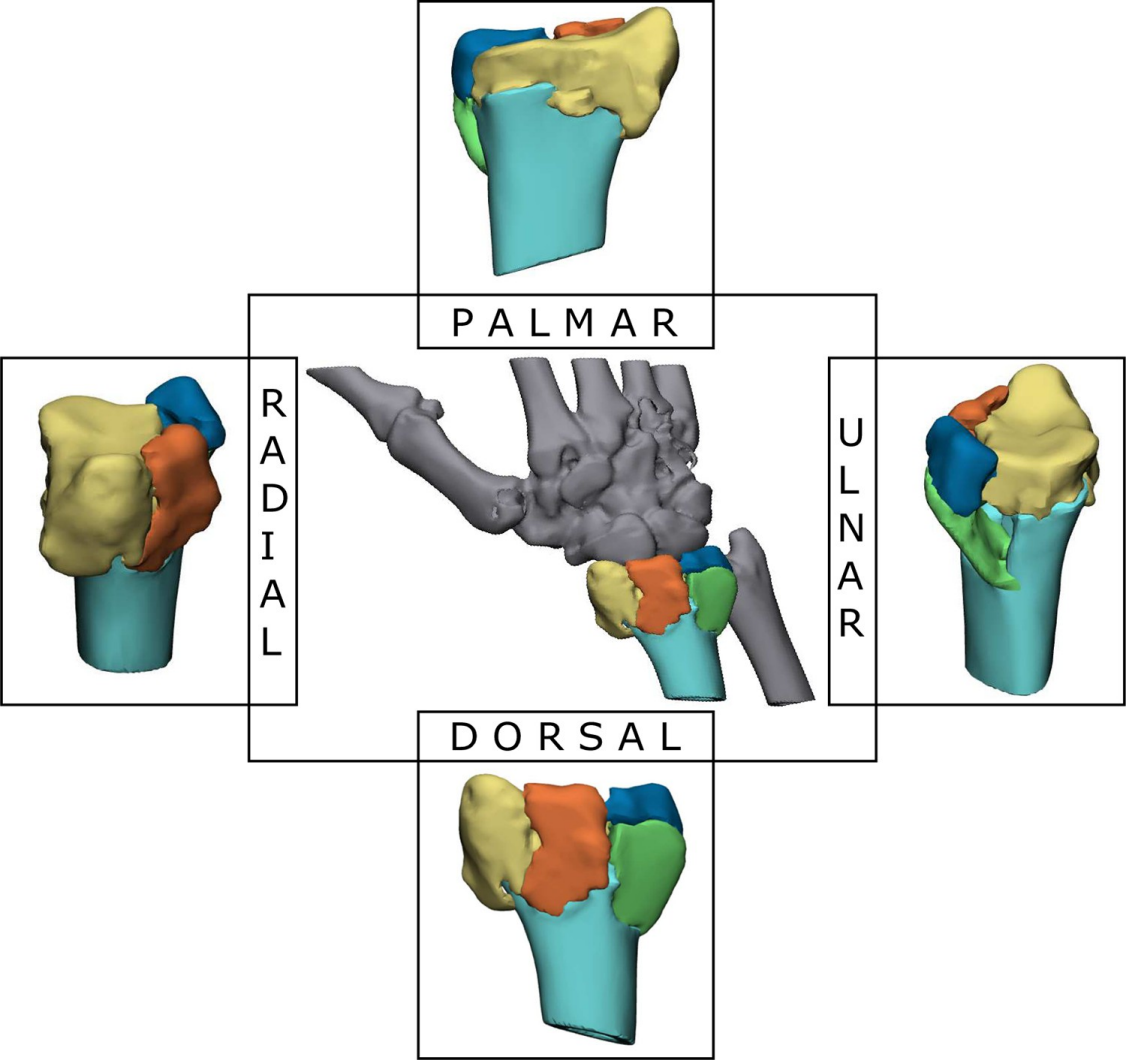
3D fracture model of the distal radius of patient 1. The central image displays a dorsal view of the fractured radius with the hand and ulna in grey for orientation. The radial shaft is displayed in light blue. The randomly assigned yellow, orange, and dark blue colors indicate intra-articular fragments. In this specific case the green fragment is considered extra-articular.

A 3D model was created, based on the original CT-data of each fracture, by using Mimics Medical software package (Version 22.0, Materialise, Leuven, Belgium) (Fig 1). CT-data (DICOM files) were imported, and the bony tissue was extracted with a threshold (Hounsfield units> 225). The region growing tool was used to remove noise and bony structures adjacent to the radius. The fracture fragments of the radius were separated manually with the split mask tool and assigned different colors.

### 3D gap area measurements

**Fig 2:**
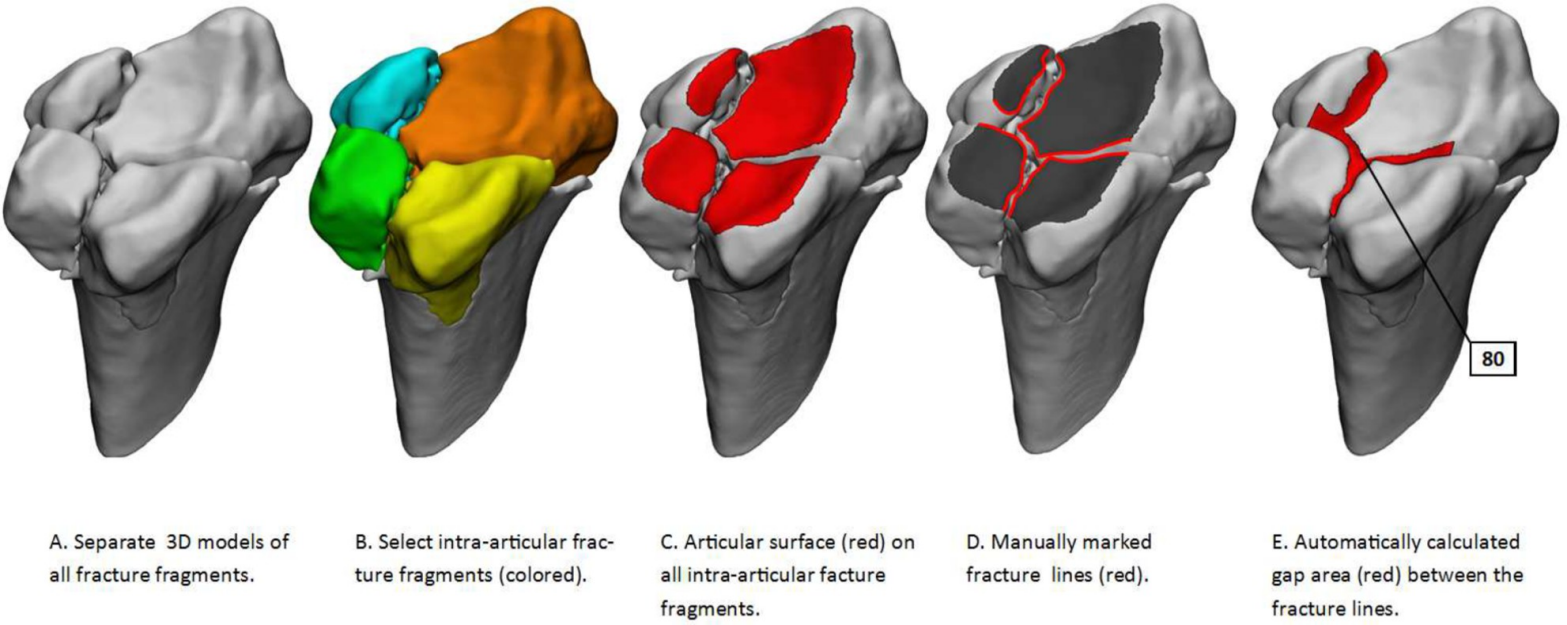
3D gap area measurement (= 80 mm^2^) illustrated on the 3D models (volar view with 45 degrees tilt towards volar/ulnar side) of patient 7. A: 3D model of the fractured wrist; B: All fragments are randomly assigned different colors (all four are intra articular in this case.); C: The articular surface is marked red on all intra-articular parts; D: Fracture lines in the articular surface are marked red; E: The gap area (red) was automatically calculated between the fracture lines.

The 3D gap area measurement was performed by three technical physicians with experience in 3D-modelling and fracture analysis (LR, AM, NA). 3D models were imported in the 3-Matic Medical package (Version 14.0, Materialise, Leuven, Belgium) (Fig 2A), and assigned colors randomly to improve fragment recognition (Fig 2B). The distal radial articular surface was manually marked on every intra-articular fracture fragment and assigned to a separate surface (Fig 2C). After marking, all articular surfaces were copied to a new part. Fracture lines were extrapolated from the articular surface contours (Fig 2D). The fracture lines were connected with the corresponding fracture lines of opposing fracture fragments. Then, a surface was created in 3-matic based on the curve representing the fracture lines (Fig 2E) and the 3D gap area was automatically calculated by 3-Matic. This process takes between 30–45 minutes depending on the complexity of the fracture.

### 2D gap and step-off measurements

**Fig 3:**
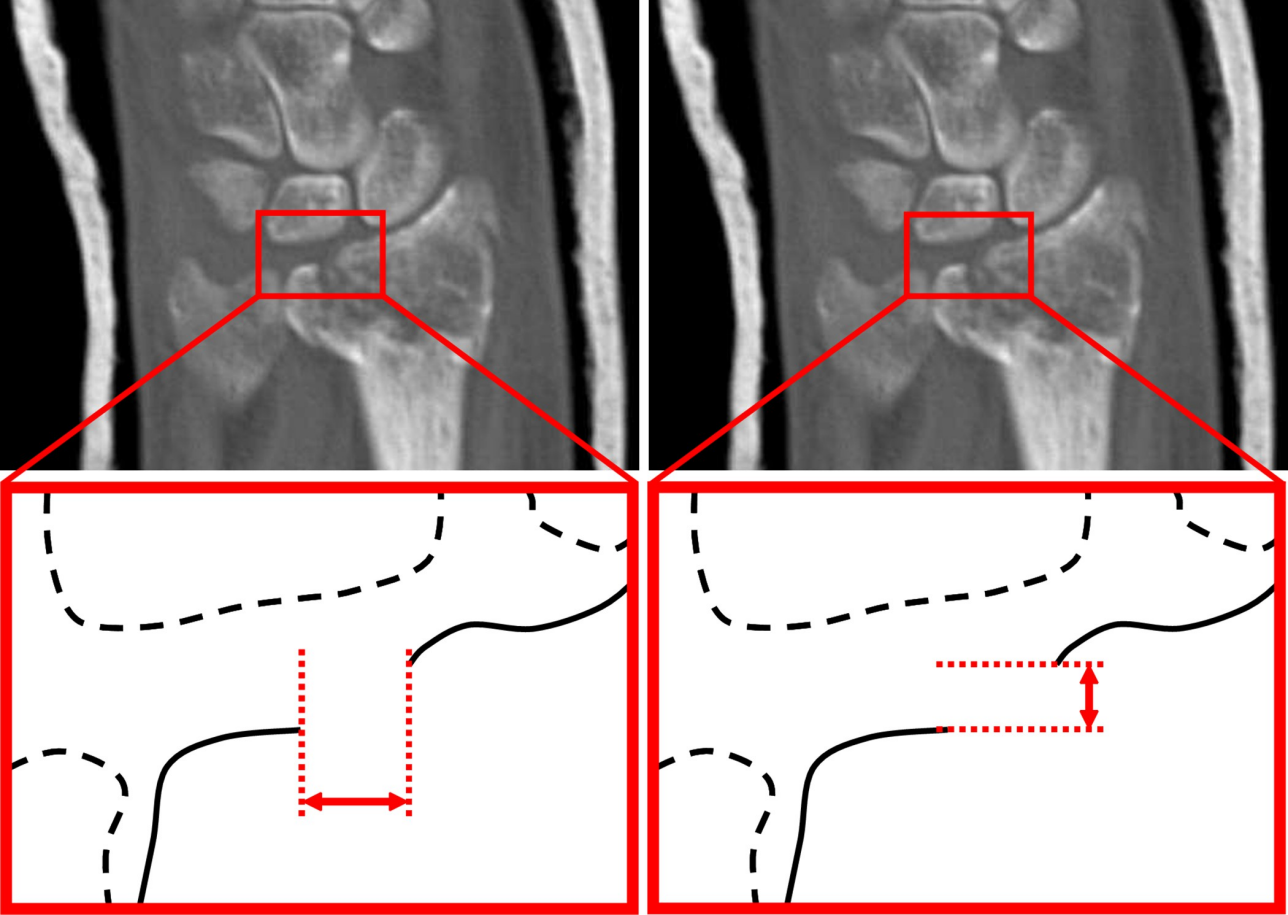
Coronal CT-slice of the wrist of patient 1 with a schematic representation of gap and step-off measurements. Left image: Measurement of the 2D gap (3.6 mm). Right image: Measurement of the 2D step-off (1.6 mm).

To compare the new 3D measurement method with the current clinical practice, conventional 2D measurements were performed on each patient by three trauma surgeons (JH, KtD, FIJ). Observers were instructed to perform measurements on three CT-slices that contained the largest gap and step-off in axial, sagittal and coronal views. The 2D gap, defined as the distance between two fracture fragments along the articular surface (illustrated in Fig 3, left image), was measured on the axial, sagittal and coronal CT-slices. Also, the 2D step-off, the largest displacement perpendicular to the articular surface (illustrated in Fig 3, right image), was measured in sagittal and coronal slices. For further analysis, only the maximal gap and step-off per observer per case was used.

### Patient-reported outcome

Both the DASH (Disability of the Arm, Shoulder and Hand Questionnaire) and the PRWE (Patient Rated Wrist Hand Evaluation) questionnaire were send to the patients by posted mail. The DASH evaluates patient complaints and influence on daily life and hobbies. The PRWE is short questionnaire that analyses pain and loss of function in the past week. All patients that were included fully completed and returned both questionnaires. Outcome of both questionnaires can range from 0–100 points, with a lower score indicating better patient-reported outcome [20,21].

### Statistics

All 3D gap area measurements were performed by three technical physicians (LR, AM, NA) with experience in 3D-modelling and fracture analysis. 2D gap and step-off measurements were performed by three trauma surgeons (JH, KtD, FIJ). To analyze inter- and intraobserver variability the intraclass correlation coefficient (ICC), with the 95% confidence interval (95% CI), was calculated in SPSS (version 26, IBM, Chicago, IL, US). Agreement was considered poor when the ICC is under 0.40, fair when between 0.40 and 0.59, good when between 0.60 and 0.74 and excellent when 0.75 or higher [22]. Also, the actual measured values of the 3D gap area and of the 2D gap and step-off were analyzed and compared between observers. Patient-reported outcome was analyzed based on the median scores of the DASH and PRWE questionnaires. The median and interquartile range (IQR) were calculated for non-normal distributed data and the mean and standard deviation for data with a normal distribution.

## Results

Forty patients with a distal radial fracture had a mean age of 57 years (range 20–85) and 33% of them were male (13/40). Three fractures were classified as AO23-B1, four as AO23-B2, three as AO23-B3, four as AO23-C1, ten as AO23-C2 and sixteen as AO23-C3.

### 2D gap and step-off measurements

Observer 1, 2 and 3 measured a median maximal gap of 2.9 (IQR: 2.3–3.3) mm, 2.6 (IQR: 1.8–3.6) mm, 2.8 (IQR: 1.8–3.4) mm respectively (S1 Table). The measured median step-off was 0.0 (IQR: 0–1.1) mm, 0.9 (IQR: 0.4–1.8) mm and 0.9 (IQR: 0–1.6) mm, respectively. The measurements of each observer for each patient can be found in the supporting information (S1 Table). Measurements as performed by observer 1 showed that six patients had a gap and step-off below 2 mm, and 34 (85%) had a gap and/or step-off above the surgical cut-off (e.g. gap and/or step-off >2 mm). In measurements of observer 2, 29 out of forty (73%) patients had a gap and/or step above 2 mm. According to observer 3, 29 out of forty (73%) patients had a gap and/or step-off above 2 mm. The interobserver agreement on the 2D measurements was fair (ICC = 0.54) for the gap and poor (ICC = 0.21) for the step-off (Table 1). The total median gap was measured at 2.8 (IQR: 1.9–3.5) mm and the step-off was 0.9 (IQR: 0.0–1.6) mm.

**Table 1:** Inter- and intraobserver measurements.

Measurements		Median (IQR*)	Median difference between observers (IQR*)	ICC* (95% CI*)
2D interobserver	Gap	2.8 (1.9–3.5) mm	0.4 (0.2–1.0) mm	0.54 (0.35–0.70)
	Step-off	0.9 (0–1.6) mm	0.6 (0.1–1.0) mm	0.21 (0.02–0.42)
3D interobserver	Gap area	36.0 (22.0–63.0) mm^2^	6.0 (2.0–19.0) mm^2^	0.81 (0.68–0.90)
3D intraobserver		36.5 (15.8–65.8) mm^2^	4.0 (1.5–5.5) mm^2^	0.98 (0.96–0.99)

*IQR = Interquartile range.

*ICC = intraclass correlation coefficient.

*95% CI = 95% confidence interval.

### 3D gap area measurements

Interobserver agreement on 3D gap area measurements was excellent: ICC = 0.81, with a median 3D gap area of 36.5 (IQR: 15.8–65.8) mm^2^ and a median difference between measurements of 6.0 (IQR: 2.0–19.0) mm^2^ (Table 1). Intraobserver agreement was also excellent: ICC = 0.98, with a median 3D gap area of 36.0 (IQR: 22.0–63.0) mm^2^ and a median difference between measurements of 4.0 (IQR: 1.5–5.5) mm^2^ (Table 1). The median 3D gap area measured by observer 1 was 36.0 (IQR: 18.0–61.8) mm^2^, 44.0 (IQR: 27.5–75.8) mm^2^ for observer 2 and 30.5 (IQR: 14.0–48.3) mm^2^ when measured by observer 3. Observer 1 performed the measurements twice and measured a median of 36.5 (IQR: 22.8–63.0) mm^2^ the second time. The measurements of each observer for each patient can be found in the supporting information (S2 and S3 Tables).

### Patient reported outcome

All patients and completed the DASH and the PRWE questionnaire at median follow-up of 77 months (range 31–119 months). The median score of the DASH was 4.2 (IQR: 0.0–10.4) and of the PRWE 5.5 (IQR: 0.0–24.4). The six patients with a gap and a step-off below the 2 mm cut-off value (according to the main observer: observer 1) had a median DASH outcome of 3.4(IQR: 0.4–8.8) and a PRWE score of 3.5 (IQR: 0.0–12.6). Clinical outcome of the 34 patients, who had a gap and/or step-off of 2 mm or larger is: DASH: 4.2 (IQR: 0.0–11.6) and a PRWE score of 5.0 (IQR: 0.0–25.5). Within this group there were three patients that had both the gap and step-off above the 2 mm cut-off and had a DASH score of 0.0, 16.7 and 68.3 and a PRWE score of 0, 41 and 37.5, respectively. All data is available in the supporting information (S4 Table).

## Discussion

The aim of this study was to introduce and validate a novel 3D measurement method to quantify the fracture displacement of intra-articular distal radius fractures. Compared to conventional 2D gap and step-off measurements, this method showed to be superior in terms of reliability. The results indicate that the 3D gap area is a reliable and reproducible measure with an inter- and intraobserver ICC of 0.81 and 0.98 respectively. It could therefore be applied in the diagnostic quantification of fracture displacement of distal radius fractures.

Conventional gap and step-off measurements are known for low reliability and reproducibility [6,8]. Kreder et al. [8] found an interobserver agreement on gap and step-off measurements to be only poor (0.35 and 0.27 respectively), and an intraobserver agreement on both measures to be poor as well (ICC = 0.37 and 0.22, respectively). Stirling et al. [6] also found a poor interobserver correlation for intra-articular gap and step-off measurements with an ICC of 0.27 and 0.31 respectively. These findings show that large deviations exist when using 2D imaging measurements on 2D CT-slices. This is consistent with our findings of a fair and poor inter- and intraobserver agreement (ICC = 0.54 and 0.21 respectively.) More literature has been dedicated to the clinical importance of the 2mm cut off of the gap and step-off. Kreder et al. concluded that the limit of 2 mm as a predictor of osteoarthritis should be abandoned, because it is an unreliable measure and predictor of outcome [8]. They also stated that the 2D gap and step-off measurements may be insufficient to quantify the multiplanar dislocations. However, no alternative has been offered in literature to improve prediction of fracture outcome or guide surgical decision-making.

Previous studies from our research group showed that 3D fracture quantification may improve outcome prediction of various fracture types. Our results have already shown better agreement on the measures with excellent interobserver agreement (ICC = 0.81), which are comparable with the agreement found in our previous studies (ICC = 0.99 and 0.94) [17,18]. Additionally, the 3D gap area fracture quantification is based on the whole intra-articular aspect of the fracture, rather than one CT-slice. Observers often selected different 2D CT-slices based on fracture insight and experience, and also selected different locations or fragments to measure the 2D gap and step-off [5]. The potential advantage of the 3D measure is that not only the size or distance of the displacement, but also the quantity (3D gap area between all fracture fragments) is incorporated in one measure.

An incidental finding is the discrepancy between the treatment performed in this group of distal radius fractures and the surgical advice according to the guidelines. It is recommended that fractures with a gap and/or step-off ≥ 2 mm should be treated surgically to achieve favorable outcome. Mulders et al. [4] show that there is a high consensus within Europe about this cut-off value. However, of the forty conservatively treated patients included in this study, a total of 34 patients should have been treated surgically according to the guidelines, because the gap and/or step-off exceeded the 2 mm cut-off. Nevertheless, the results from the DASH and PRWE questionnaires after nonoperative treatment show that these patients report good outcomes, despite treatment deviation from the guideline. Therefore, it is questionable whether the 2D gap and step-off measurement is discriminative for functional outcome. The potential advantage of 3D gap area measurements is that is represents the displacement of the entire fracture and can be used as a standardized quantitative measure of the extent of the fracture.

An important limitation of this study is the software and expertise required to perform the 3D gap area measurements. The software may not be available in all hospitals. Also, the technical expertise that is required to create and measure 3D models may not yet be available everywhere, and also does not eliminate all interobserver variability in segmentation and measurements. A second limitation is that this 3D analysis is not available in acute situations as it is more time consuming that the simple x-ray or CT-scan evaluation. Technical physicians must be available to create models and perform measurements take 30–60 minutes per case. Automatization of the CT-scan segmentation and implementation of the 3D models and measurements in the electronic patient record may facilitate the process of clinical application in the near future. These steps could also decrease the inter- and intraobserver variability even further. In our hospital this method is currently used in addition to the 2D measurements of distal radius fractures. The 3D view is considered when creating an operative plan and 3D gap area measurements are used to estimate fracture impact.

In the future, the 3D gap area could be seen as a potential addition to distal radius fracture quantification. Before the method can be fully incorporated, the segmentation and measurement process should be automatized to eliminate interobserver variability. The results indicate that fractures with a 3D gap area below 150 mm^2^ may result in a good patient-reported outcome, if they are treated non-surgically. However, the current patient group of forty cases is too small to correlate measurements to patient-reported outcome measures. Therefore, in order to find a good cut-off value for the 3D gap area a follow-up study will have to be performed with the focus on linking the 3D gap area to patient-reported outcomes in a larger patient series.

In conclusion, the 3D gap area is a less observer dependent alternative to the conventional gap and step-off measurements for assessing fracture displacement of distal radius fractures. The method can be used in addition to the currently used classification of distal radius fractures, especially in fractures which have a gap and/or step-off around the surgical 2 mm cut-off.

## Supporting information

S1 Table Interobserver measurements of the 2D gap and step-off. *Obs. = Observer, *IQR = Interquartile range.
(PDF)

S2 Table Interobserver data 3D. Exact measures of the 3D gap area per case (1–20) measured by all three observers. The median difference is the median of the difference between all three observers. *IQR = Interquartile range.
(PDF)

S3 Table Intraobserver data 3D. Exact measures of the 3D gap area per case (1–20) measured twice by observer 1. The difference is the difference between the two measurements. *IQR = Interquartile range.
(PDF)

S4 Table Patient reported outcome: DASH and PRWE. Compared to 2D gap and/or step (Obs 1). *Obs. = Observer, *IQR = Interquartile range.
(PDF)

## References

[1] Nederlandse vereniging voor Heelkunde. Richtlijn distale radius fracturen: diagnostiek en behandeling. 2010. (Dutch).

[2] Kennisinstituut van de Federatie van Medisch Specialisten. Distale radius fracturen. 2012. Dutch.

[3] Meinberg E, Agel J, Roberts C, Karam M, Kellam J. Fracture and Dislocation Classification Compendium—2018. Journal of Orthopaedic Trauma. 2018;32. doi: 10.1097/BOT.0000000000001063 29256945

[4] Mulders MA, Rikli D, Goslings J, Schep N. Classification and treatment of distal radius fractures: a survey among orthopaedic trauma surgeons and residents. European Journal of Trauma and Emergency Surgery. 2017;43(2):239–48. doi: 10.1007/s00068-016-0635-z 26872680 PMC5378748

[5] Meesters AM, Ten Duis K, Banierink H, Stirler VM, Wouters PC, Kraeima J, et al. What are the interobserver and intraobserver variability of gap and stepoff measurements in acetabular fractures? Clinical Orthopaedics and Related Research®. 2020;478(12):2801–8. doi: 10.1097/CORR.0000000000001398 32769535 PMC7899427

[6] Stirling E, Jeffery J, Johnson N, Dias J. Are radiographic measurements of the displacement of a distal radial fracture reliable and reproducible? The bone & joint journal. 2016;98(8):1069–73. doi: 10.1302/0301-620X.98B8.37469 27482019

[7] Cole RJ, Bindra RR, Evanoff BA, Gilula LA, Yamaguchi K, Gelberman RH. Radiographic evaluation of osseous displacement following intra-articular fractures of the distal radius: reliability of plain radiography versus computed tomography. The Journal of hand surgery. 1997;22(5):792–800. doi: 10.1016/s0363-5023(97)80071-8 9330135

[8] Kreder HJ, Hanel DP, McKee M, Jupiter J, McGillivary G, Swiontkowski MF. X-ray film measurements for healed distal radius fractures. The Journal of hand surgery. 1996;21(1):31–9. doi: 10.1016/S0363-5023(96)80151-1 8775193

[9] Belloti JC, Tamaoki MJS, Franciozi CE, Dos Santos JBG, Balbachevsky D, Chapchap E, et al. Are distal radius fracture classifications reproducible? Intra and interobserver agreement. Sao Paulo Medical Journal. 2008;126:180–5. doi: 10.1590/s1516-31802008000300008 18711658 PMC11026013

[10] Jin W-J, Jiang L-S, Shen L, Lu H, Cui Y-M, Zhou Q, et al. The interobserver and intraobserver reliability of the cooney classification of distal radius fractures between experienced orthopaedic surgeons. The Journal of Hand Surgery: European Volume. 2007;32(5):509–11. 10.1016/j.jhse.2007.03.002 17950210

[11] Naqvi S, Reynolds T, Kitsis C. Interobserver reliability and intraobserver reproducibility of the Fernandez classification for distal radius fractures. Journal of Hand Surgery (European Volume). 2009;34(4):483–5. doi: 10.1177/1753193408101667 19321533

[12] Ploegmakers JJ, Mader K, Pennig D, Verheyen CC. Four distal radial fracture classification systems tested amongst a large panel of Dutch trauma surgeons. Injury. 2007;38(11):1268–72. doi: 10.1016/j.injury.2007.03.032 17643439

[13] Van Leerdam RH, Souer JS, Lindenhovius AL, Ring DC. Agreement between initial classification and subsequent reclassification of fractures of the distal radius in a prospective cohort study. Hand. 2010;5(1):68–71. doi: 10.1007/s11552-009-9212-9 19588208 PMC2820616

[14] Mellema JJ, Janssen SJ, Guitton TG, Ring D. Quantitative 3-dimensional computed tomography measurements of coronoid fractures. The Journal of hand surgery. 2015;40(3):526–33. doi: 10.1016/j.jhsa.2014.07.059 25510153

[15] Lubberts B, Janssen S, Mellema J, Ring D. Quantitative 3-dimensional computed tomography analysis of olecranon fractures. Journal of shoulder and elbow surgery. 2016;25(5):831–6. doi: 10.1016/j.jse.2015.10.002 26711473

[16] De Muinck Keizer R-JO, Meijer DT, van der Gronde BA, Teunis T, Stufkens SA, Kerkhoffs GM, et al. Articular gap and step-off revisited: 3D quantification of operative reduction for posterior malleolar fragments. Journal of orthopaedic trauma. 2016;30(12):670–5. doi: 10.1097/BOT.0000000000000676 27479735

[17] Meesters A, Kraeima J, Banierink H, Slump C, De Vries J, Ten Duis K, et al. Introduction of a three-dimensional computed tomography measurement method for acetabular fractures. PloS one. 2019;14(6):e0218612. doi: 10.1371/journal.pone.0218612 31216346 PMC6583999

[18] Assink N, Kraeima J, Slump CH, ten Duis K, de Vries JPPM, Meesters AML, et al. Quantitative 3D measurements of tibial plateau fractures. Scientific Reports. 2019;9(1):14395. 31591466 10.1038/s41598-019-50887-6 PMC6779915

[19] Brouwers L, Pull ter Gunne AF, de Jongh MAC, van der Heijden FHWM, Leenen LPH, Spanjersberg WR, et al. The Value of 3D Printed Models in Understanding Acetabular Fractures. 3D Printing and Additive Manufacturing. 2018;5(1):37–46.

[20] Hudak PL, Amadio PC, Bombardier C. Development of an upper extremity outcome measure: the DASH (disabilities of the arm, shoulder and hand) [corrected]. The Upper Extremity Collaborative Group (UECG). American Journal Industrial Medicine. 1996;29(6):602–608. doi: 10.1002/(SICI)1097-0274(199606)29:6&lt;602::AID-AJIM4&gt;3.0.CO;2-L 8773720

[21] MacDermid JC, Turgeon T, Richards RS, Beadle M, Roth JH. Patient rating of wrist pain and disability: a reliable and valid measurement tool. Journal of Orthopaedic Trauma. 1998;12(8):577–586. doi: 10.1097/00005131-199811000-00009 9840793

[22] Cicchetti DV. Guidelines, criteria, and rules of thumb for evaluating normed and standardized assessment instruments in psychology. Psychological assessment. 1994;6(4):284.

