# Supplementary material for: A new quantitative 3D gap area measurement of fracture displacement of intra-articular distal radius fractures: Reliability and clinical applicability
Source: PLoS One. 2022 Sep 27;17(9):e0275206. doi: 10.1371/journal.pone.0275206 (PMC9514643; doi:10.1371/journal.pone.0275206)
Supplement: S1 Table — *Obs. = Observer, *IQR = Interquartile range. (PDF) [file pone.0275206.s001.pdf]

**Case**	**Gap**				**Step-off**			
	**Obs.*1**	**Obs.*2**	**Obs.*3**	**Median difference**	**Obs.*1**	**Obs.*2**	**Obs.*3**	**Median difference**
**1**	2.9	1.9	1.6	**1.0**	0.0	0.0	0.0	**0.0**
**2**	1.8	1.4	1.6	**0.2**	1.8	0.9	1.4	**0.5**
**3**	2.9	2.1	2.8	**0.7**	0.0	2.0	1.8	**1.8**
**4**	3.5	2.2	1.4	**1.3**	0.0	1.2	0.6	**0.6**
**5**	2.9	3.1	2.9	**0.2**	0.0	2.3	1.8	**1.8**
**6**	3.0	3.3	1.2	**1.8**	2.3	1.7	2.7	**0.6**
**7**	3.8	3.9	7.8	**3.9**	0.0	0.0	1.9	**1.9**
**8**	1.8	1.5	2.0	**0.3**	0.0	0.8	0.8	**0.8**
**9**	4.5	4.4	4.7	**0.2**	0.0	0.9	0.0	**0.9**
**10**	2.0	0.8	1.0	**1.0**	0.0	0.5	0.0	**0.5**
**11**	3.0	4.7	3.3	**1.4**	1.6	0.9	0.0	**0.9**
**12**	3.4	4.1	1.9	**1.5**	0.0	0.9	0.0	**0.9**
**13**	3.9	1.8	4.6	**2.1**	5.5	0.0	0.0	**5.5**
**14**	4.5	3.5	4.2	**0.7**	1.6	2.0	1.7	**0.3**
**15**	2.5	3.5	2.5	**1.0**	1.0	2.6	1.7	**0.9**
**16**	3.2	3.5	3.3	**0.2**	0.0	0.0	0.0	**0.0**
**17**	2.7	2.6	1.7	**0.9**	1.0	1.4	0.0	**1.0**
**18**	4.1	4.0	3.1	**0.9**	0.5	0.5	0.0	**0.5**
**19**	1.8	1.4	1.0	**0.4**	0.0	0.0	0.0	**0.0**
**20**	3.0	3.3	3.2	**0.2**	0.0	2.2	0.0	**2.2**
**21**	4.4	2.6	2.7	**1.7**	0.0	2.2	1.0	**1.3**
**22**	2.6	3.9	2.9	**1.0**	2.2	0.0	2.3	**2.2**
**23**	3.8	6.5	3.8	**2.7**	0.0	0.0	0.0	**0.0**
**22**	2.5	4.8	3.8	**1.3**	1.0	2.0	1.6	**0.6**
**25**	2.7	3.5	2.7	**0.8**	1.0	1.4	1.0	**0.4**
**26**	2.9	3.0	2.7	**0.2**	0.0	0.5	0.0	**0.5**
**27**	3.6	5.7	3.8	**1.9**	1.6	2.6	1.8	**0.8**
**28**	2.9	3.3	3.0	**0.3**	1.0	2.2	1.4	**0.8**
**29**	2.2	1.8	1.8	**0.4**	0.0	1.2	0.0	**1.2**
**30**	1.7	1.4	1.1	**0.3**	0.0	1.0	0.9	**0.9**
**31**	0.9	0.9	1.0	**0.1**	2.1	2.0	1.2	**0.8**
**32**	2.8	2.5	3.4	**0.6**	1.2	1.2	0.5	**0.7**
**33**	1.8	1.8	1.8	**0.0**	0.0	0.0	1.1	**1.1**
**34**	2.8	1.8	2.8	**1.0**	0.0	0.9	0.0	**0.9**

**35**	3.1	4.1	3.5	**0.6**	1.1	0.7	3.0	**1.9**
**36**	1.7	2.1	2.4	**0.4**	0.0	0.0	1.6	**1.6**
**37**	3.0	1.8	3.5	**1.2**	1.7	1.1	1.6	**0.5**
**38**	2.0	2.6	4.6	**2.0**	0.0	0.0	1.3	**1.3**
**39**	2.6	2.2	2.4	**0.2**	0.0	0.7	0.0	**0.7**
**40**	2.3	2.2	2.2	**0.1**	0.9	1.2	0.8	**0.3**
**Median (IQR)**	**2.9 (2.3-3.3)**	**2.63 (1.8-3.6)**	**2.8 (1.8-3.4)**	**0.8 (0.3-1.3)**	**0.0 (0-1.1)**	**1.2 (0.9-2.1)**	**0.9 (0.0-1.6)**	**0.8 (0.5-1.2)**
