# Supplementary material for: A new quantitative 3D gap area measurement of fracture displacement of intra-articular distal radius fractures: Reliability and clinical applicability
Source: PLoS One. 2022 Sep 27;17(9):e0275206. doi: 10.1371/journal.pone.0275206 (PMC9514643; doi:10.1371/journal.pone.0275206)
Supplement: S2 Table — Exact measures of the 3D gap area per case (1–20) measured by all three observers. The median difference is the median of the difference between all three observers. *IQR = Interquartile range. (PDF) [file pone.0275206.s002.pdf]

Case	Observer 1 (mm2)	Observer 2 (mm2)	Observer 3 (mm2)	Median difference (mm2)
**1**	50	55	46	**4**
**2**	42	75	44	**29**
**3**	73	92	64	**23**
**4**	34	139	31	**96**
**5**	44	37	42	**5**
**6**	36	53	34	**16**
**7**	75	78	71	**5**
**8**	119	145	123	**20**
**9**	47	56	33	**14**
**10**	32	45	33	**12**
**11**	43	43	45	**2**
**12**	38	38	26	**12**
**13**	29	37	29	**4**
**14**	31	52	30	**22**
**15**	37	39	33	**4**
**16**	0	0	6	**6**
**17**	24	31	23	**8**
**18**	103	126	106	**10**
**19**	81	83	77	**4**
**20**	12	15	11	**4**
**21**	14	11	9	**5**
**22**	0	0	0	**0**
**23**	90	104	88	**2**
**22**	76	35	0	**40**
**25**	0	0	0	**0**
**26**	35	40	30	**9**
**27**	0	0	0	**0**
**28**	58	75	55	**14**
**29**	37	54	17	**37**
**30**	7	17	6	**5**
**31**	24	32	25	**6**
**32**	187	134	92	**51**
**33**	165	148	81	**67**
**34**	19	14	14	**5**
**35**	0	2	3	**2**
**36**	97	112	93	**11**
**37**	15	16	14	**1**
**38**	15	33	15	**11**
**39**	29	48	25	**19**
**40**	36	47	46	**6**
**Median (IQR)**	**36 (18-62)**	**44 (28-76)**	**31 (14-48)**	**4 (1-5)**
