# Supplementary material for: A new quantitative 3D gap area measurement of fracture displacement of intra-articular distal radius fractures: Reliability and clinical applicability
Source: PLoS One. 2022 Sep 27;17(9):e0275206. doi: 10.1371/journal.pone.0275206 (PMC9514643; doi:10.1371/journal.pone.0275206)
Supplement: S3 Table — Exact measures of the 3D gap area per case (1–20) measured twice by observer 1. The difference is the difference between the two measurements. *IQR = Interquartile range. (PDF) [file pone.0275206.s003.pdf]

Case	Observer 1 (mm2)	Observer 1.2 (mm2)	Difference (mm2)
**1**	50	53	**3**
**2**	42	46	**4**
**3**	73	69	**4**
**4**	34	43	**9**
**5**	44	48	**4**
**6**	36	37	**1**
**7**	75	73	**2**
**8**	119	125	**6**
**9**	47	57	**10**
**10**	32	33	**1**
**11**	43	42	**1**
**12**	38	43	**5**
**13**	29	33	**4**
**14**	31	29	**2**
**15**	37	35	**2**
**16**	0	0	**0**
**17**	24	22	**2**
**18**	103	116	**13**
**19**	81	80	**1**
**20**	12	23	**11**
**21**	14	18	**4**
**22**	0	0	**0**
**23**	90	106	**16**
**22**	76	75	**1**
**25**	0	0	**0**
**26**	35	31	**4**
**27**	0	36	**36**
**28**	58	61	**3**
**29**	37	13	**24**
**30**	7	12	**5**
**31**	24	26	**2**
**32**	187	185	**2**
**33**	165	184	**19**
**34**	19	24	**5**
**35**	0	0	**0**
**36**	97	101	**4**
**37**	15	15	**0**
**38**	15	22	**7**
**39**	29	29	**0**
**40**	36	41	**5**
**Median (IQR*)**	**36 (18-62)**	**37 (23-63)**	**4 (1-5)**
