# Supplementary material for: A new quantitative 3D gap area measurement of fracture displacement of intra-articular distal radius fractures: Reliability and clinical applicability
Source: PLoS One. 2022 Sep 27;17(9):e0275206. doi: 10.1371/journal.pone.0275206 (PMC9514643; doi:10.1371/journal.pone.0275206)
Supplement: S4 Table — Compared to 2D gap and/or step (Obs 1). *Obs. = Observer, *IQR = Interquartile range. (PDF) [file pone.0275206.s004.pdf]

Case	DASH	PRWE	2D gap Obs.* 1 (mm)	2D step-off Obs. * 1 (mm)
**1**	0	0	2.9	0.0
**2**	1.7	0	1.8	1.8
**3**	0.8	0	2.9	0.0
**4**	10	25.5	3.5	0.0
**5**	4.2	6	2.9	0.0
**6**	0	0	3.0	2.3
**7**	1.7	1	3.8	0.0
**8**	10	7	1.8	0.0
**9**	0	0	4.5	0.0
**10**	0	0	2.0	0.0
**11**	5	4.5	3.0	1.6
**12**	0	0	3.4	0.0
**13**	16.7	41	3.9	5.5
**14**	4.2	7	4.5	1.6
**15**	16.4	38	2.5	1.0
**16**	8.3	17	3.2	0.0
**17**	0	0	2.7	1.0
**18**	0	0	4.1	0.5
**19**	70	86	1.8	0.0
**20**	4.3	9	3.0	0.0
**21**	45	37.5	4.4	0.0
**22**	68.3	37.5	2.6	2.2
**23**	4.2	1.5	3.8	0.0
**22**	0	0	2.5	1.0
**25**	16.7	40	2.7	1.0
**26**	32.1	41	2.9	0.0
**27**	8.3	6	3.6	1.6
**28**	1.7	1	2.9	1.0
**29**	0	0	2.2	0.0
**30**	0	0	1.7	0.0
**31**	2.5	6	0.9	2.1
**32**	32.5	22	2.8	1.2
**33**	5	14.5	1.8	0.0
**34**	0	0	2.8	0.0
**35**	11.6	75	3.1	1.1
**36**	0	0	1.7	0.0
**37**	24.1	33	3.0	1.7
**38**	9.2	5	2.0	0.0
**39**	10	24	2.6	0.0
**40**	2.5	3.5	2.3	0.9
**Median (IQR*)**	**4.2 (0.0-10.4)**	**5.5 (0.0-24.4)**	**2.9 (2.3-3.3)**	**0.0 (0-1.1)**
